# Archives of Dermatology

**Published:** 1874-11

**Authors:** 


*Archives of Dermatology. A Quarterly Journal of Skin and Venereal Diseases.* Edited by L. DUNCAN BULKLEY, A. M., M. D. Vol. 1, No. 1; October, 1874.

When the announcement was made by Messrs. G. P. Putnam's Sons, that they wonld publish on October 1st, the first number of a Quarterly Journal of Dermatology, to be edited by Dr. Bulkley, we anticipated an excellent, well arranged and well conducted Journal, and we were not disappointed in the first number, now before us. It consists of ninety-six pages of matter arranged under suitable departments, each under the care of gentlemen who have distinguished themselves in their several fields.

The contents of the Journal are arranged under the following heads: Original Communications, Transactions of the New York Dermatological Society, Clinical Reports, Extracts and Translations, Digest of Dermatological Literature, Reviews and Book Notices, Correspondence and Miscellanies. The contents of the present number are of an interesting and instructive nature Among the Griginal Communications we notice articles upon Rotheln, by J. Lewis Smith, M. D., Urethral Stricture, by F. J. Bumstead, M. D., Elephantiasis of the Penis, by Robert F. Wier, M. D., and others of value. The Digest of Dermatological Literature is a valuable feature of the Journal, and the various subjects are ably reviewed by the gentlemen having charge of this department. We wish for the new Journal long life and a hearty support.

